# Turbidity in Combined Sewer Sewage: An Identification of Stormwater Detention Tanks

**DOI:** 10.3390/ijerph17093053

**Published:** 2020-04-28

**Authors:** Yang Liu, Liangang Hou, Wei Bian, Banglei Zhou, Dongbo Liang, Jun Li

**Affiliations:** 1 The College of Architecture and Civil Engineering, Beijing University of Technology, Beijing 100124, China; 2 China Energy Investment Corporation, Beijing 100011, China

**Keywords:** combined sewer system, correlation, stormwater detention tank, turbidity

## Abstract

Combined sewer overflow remains a major threat to surface water quality. A stormwater detention tank is an effective facility to control combined sewer overflow. In this study, a new method for the selective collection of combined sewer sewage during wet weather based on real-time turbidity control is established to reduce the load of pollutants entering a river using a stormwater detention tank with a limited volume. There was a good correlation found between turbidity and the concentrations of total suspended solids (TSS) (R^2^ = 0.864, *p* < 0.05), total phosphorus (TP) (R^2^ = 0.661, *p* < 0.01), and chemical oxygen demand (COD) (R^2^ = 0.619, *p* < 0.01). This study shows that turbidity can be used to indicate the concentration of TSS, TP, and COD in the sewage of the combined sewer systems in wet weather. Based on the adopted first flush detection approach, total nitrogen (TN) and TP showed the first flush effect, whereas the first flush effect of TSS and COD was not obvious. The results show that it is impossible to effectively control combined sewer overflow by only treating the initial rainwater.

## 1. Introduction

In many large cities all over the world, the combined sewer system is mostly used in old urban areas to receive rainwater runoff, domestic sewage, and industrial wastewater and is a common method of wastewater transportation [1]. Under sufficient rainfall, combined sewer overflow (CSO) occurs when sewage in the combined sewer exceeds the capacity of the wastewater treatment plants (WWTPs) [2,3,4]. In CSO events, the discharge of untreated domestic sewage and rainwater into surface water means that numerous nutrients, suspended solids, heavy metals, and pathogenic microorganisms are imported. This phenomenon has become one of the main causes of pollution for many receiving water bodies around the world [5,6,7].

It is not easy to reconstruct a combined sewer system in large cities. The construction of stormwater detention tanks is considered to be an effective measure to reduce the negative impact of CSO [8,9]. However, due to factors such as floor space and construction investments, some WWTPs in China are not equipped with stormwater detention tanks of sufficient capacity. Reducing CSO as much as possible using limited storage volume has become a serious problem in the treatment of non-point pollution in China.

At present, most detention tanks are designed and constructed based on information such as catchment area, rainfall depth, rainfall time, and pipe network system [10,11]. Nevertheless, Suarez [12] carried out the monitoring and analysis of the confluence areas of five different cities in Spain. All the catchment areas showed no significant pollution first flush effect, and the main pollution load of a rainfall event was discretely distributed. Similarly, the results of Rauch [13] showed that reducing the discharge of wastewater in wet weather may not necessarily improve the water quality of the receiving water body, which raised doubts about the effect of using only the volumetric method to control the pollution of combined sewer overflow. Rathnayake [14] found that relying solely on rainfall depth control can increase the design volume of the stormwater detention tank and cannot reasonably reduce overflow pollution from the combined system. The operating state of a detention tank is regulated based on rainfall data, which are relatively one-sided, especially when the concentration of influent pollutants is low. Studies have shown that the initial rainfall depth is large, but the first flush effect of pollutants is not obvious [15,16,17]. In this case, if the concentration of pollutants is very high in the later period, due to insufficient volume, the storage facilities will have little effect on the removal of pollutants, which will cause great harm to the receiving water. Therefore, Stormwater detention tanks based on water quality judgment should be developed, rather than rainfall variables. However, there are relatively few studies on the selective collection of combined sewer sewage based on the high frequency turbidity measurements used to estimate the concentration of pollutants.

In this study, water samples from 21 typical rainfall events from 2017 to 2019 were collected in the southeast area of Beijing, and the water quality characteristics of the combined wastewater were measured and analyzed. The correlations between turbidity and the primary pollutant concentrations in the combined sewer sewage, including total phosphorus (TP), total suspended solids (TSS), chemical oxygen demand (COD), and total nitrogen (TN), were investigated. Using the information on the concentration of primary pollutants provided by turbidity measurements, heavily polluted sewage can be retained selectively in stormwater detention tanks, while lightly polluted sewage is preferably supposed to be discharged into the receiving water. In this way, the volume of the storage tank was used effectively, and the pollution load of the receiving water during wet weather was reduced.

## 2. Materials and Methods

### 2.1. Study Site

The examined areas located in Tongzhou District and Chaoyang District covered 37.09 and 46.42 ha, respectively, and the impervious area fractions were 32.5% and 67.8%, respectively. This is an important diplomatic and commercial area of Beijing and a sub-central area of the city under vigorous construction. The average annual rainfall in Beijing is 585 mm, and most of the rainfall occurs in April to October, accounting for more than 85% of the total annual rainfall. 

**Figure 1:**
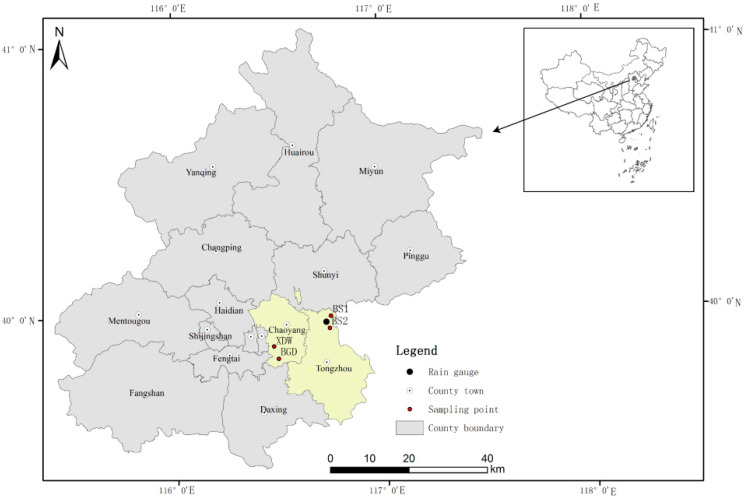
Locations of the four monitoring points in the southeast area of Beijing.

Four monitoring points were selected in this study: BS1, BS2, BGD, and XDW. The specific locations are shown in Figure 1, and all are located in the combined sewers before entering the wastewater treatment facilities or WWTPs. 

### 2.2. Sampling and Sensors

To establish the correlation functions between turbidity and the primary pollutants, 25 sampling sessions were performed between 2017 and 2019 at the study site. Four were conducted during dry weather, and the remaining 21 were performed during different rainfall events. The four monitoring points were equipped with turbidimeters and flow meters. The turbidity sensor is a Viso Turb 700 IQ electrode (WTW, Munich, Germany), which is used to measure turbidity in the range of 0–4000 NTU (nephelometric turbidity unit). Every 5 min, the average turbidity value is calculated by the integration of 15 s of data. On-site calibration is checked using kieselguhr [18], and no sensor drift was observed during the experiment. At the monitoring point, a flow meter (Hydreka, Lyon, France) based on the acoustic Doppler principle recorded the flow, and the rainfall information was monitored by a tipping bucket rain gauge located in the Tongzhou study area, as shown in Figure 1. 

In order to measure the other water quality parameters (except for turbidity), a sample was collected in the combined sewer every 5 min at the beginning of the rainfall, and the interval of the later sampling was 10–15 min. The specific operation depended on the intensity of the rainfall. Samples were transported to the laboratory in polyethylene bottles and stored in a refrigerator at 4 ℃ before testing.

### 2.3. Analytical Methods

The NH_4_^+^-N, TSS, COD, and TP analyses of wastewater were performed in accordance with the American Public Health Association (APHA) Standard [19]. TN was measured by a vario TOC-TN instrument. In particular, TN included the total kjeldahl nitrogen, NO_3_^–^-N, and NO_2_^–^-N. Dissolved oxygen (DO), pH, and temperature were monitored by a WTW Multi 3420i meter (WTW, Munich, Germany).

All water quality data were analyzed using Microsoft Excel, Origin, and SPSS. A one-way analysis of variance was used to analyze the treatment effect. The SPSS 24.0 software (IBM, Armonk, NY, USA) was used for a linear regression analysis and curve fitting. The p value was less than 0.05, indicating that the two pairs had good statistical significance.

### 2.4. Data Treatment

Concentrations of pollutants often change greatly during the same rainfall process. The use of the event mean concentration (EMC) can better characterize the pollution characteristics of rainfall events [12]. The EMC represents the flow-weighted average concentration computed as the total pollutant mass during a rainfall event divided by the total flow volume of the combined sewer [20] and is expressed as:

(1) $$EMC=\frac{M}{V}=\frac{\int_{0}^{t_{r}}C_{t}\cdot Q_{t}dt}{\int_{0}^{t_{r}}Q_{t}dt}≅\frac{\sum C_{t}\cdot Q_{t}\Delta t}{\sum Q_{t}\cdot \Delta t}$$

where EMC (mg/L) is the event mean concentration, M (g) is the total mass of the pollutant over the entire rainfall event duration, V (m^3^) is the total volume of sewage over the entire rainfall event, Ct (mg/L) is the time variable concentration, Qt (m^3^/min) is the time variable flow and Δt (min) is the sampling interval, t_r_ is the total time of the entire rain event, and t is each sampling time.

In order to quantitatively analyze the degree of the first flush effect, Bertrand [21] proposed data fitting the measured dimensionless M (v) curve. The size of the first flush is characterized by the size of the fitting index b, and the four intervals are defined according to the size of the initial scour coefficient. The procedures presented above imply a subjective/arbitrary estimation of the first flush volume [22], but the main purpose of this study is to identify heavily polluted sewage, so this commonly used method is still used to quantitatively determine the first flush effect.

## 3. Results and Discussion

### 3.1. Combined Sewer Sewage Quality

**Figure 2:**
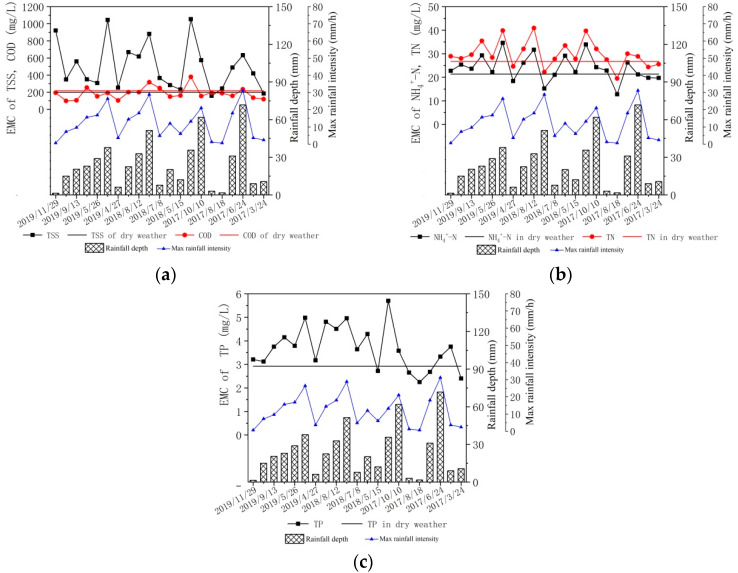
Rainfall depth, max rainfall intensity, and event mean concentration (EMC) of primary pollutants in 21 storm events. The horizontal line represents the dry weather concentration of the corresponding pollutant. (**a**): The EMC of total suspended solids (TSS) and chemical oxygen demand (COD); (**b**): The EMC of NH_4_^+^-N and total nitrogen (TN); (**c**): The EMC of total phosphorus (TP).

This study measured five pollutant indicators (NH_4_^+^-N, TN, TP, COD, and TSS), electrical conductivity, and turbidity during dry weather and 21 rainfall events at four monitoring points in the southeast area of Beijing. Using Formula (1), the EMC value of each pollutant for each rainfall event was calculated (Figure 2). The ammonium nitrogen in the combined sewer sewage in wet weather accounted for 76.5–93.2% of the total nitrogen. The average concentrations of NH_4_^+^-N, TN, TP, COD, and TSS pollutants in the 21 rainfall events were 15.50, 20.69, 3.25, 142.3, and 313.3 mg/L, respectively, which are higher than the concentration limits of class V water bodies in the “Environmental quality standards for surface water” (GB3838-2002). Serious pollution of receiving water will occur unless the sewage during wet weather is collected and treated. Therefore, appropriate measures should be taken to control sewage under these conditions.

The combined sewer sewage in wet weather was mainly composed of surface runoff, domestic sewage, and sewer sediment [23]. In this study, the EMC of COD, NH_4_^+^-N, and TN in the 21 rainfall events fluctuated around the concentration of the dry weather pollutants. This is different from previous studies in which the flow of the combined sewer system increased during wet weather, while the pollutant load decreased [16,24]. In this study, the inflow of the combined sewer increased during wet weather, but the concentrations of TSS and TP increased (Figure 2a,c), possibly because the impact of suspended solids and pollutants washed away from the ground on the concentration of pollutants in the sewer was greater than the dilution of the rainwater. For dissolved pollutants such as NH_4_^+^-N, TN, and COD, because the contribution of rainfall runoff was small, the contribution of sediment pollution in the sewer and the dilution of rainwater worked together, making the pollutant concentration fluctuate during dry weather (Figure 2a,b).

### 3.2. Pollutant First Flush in Combined Sewer

The first flush effect refers to the phenomenon when, during a rainfall, the initial sewage carries most of the pollution load [16]. The existence of the first flush effect involves the scale and investment in combined sewer overflow control facilities, such as stormwater detention tanks. If the first flush effect can be accurately identified, a treatment system can be designed to store and treat the most polluted parts of the sewage, while directly bypassing the remaining minimally processed or unprocessed portions, which has important research significance [17,25]. The monitored rainfall events have different rainfall depths and sewage volume. The adopted “first flush” procedure has a dimensionless nature that disregards the impact of storm volume [22,26]. However, the main purpose of this study is to selectively collect heavily polluted sewage. Therefore, the first flush parameter b was used to quantitatively characterize the pollutant first flush effect. The fitting formula used is shown in Formula (2):

(2) $$Y=X^{b}$$

where Y is the cumulative pollutant mass rate, X is the cumulative sewage volume rate, and b is the fitting parameter, which is the first flush coefficient. The smaller the b value, the stronger the pollutant first flush effect, and vice versa. According to the 30/80 standard proposed by Deletic and Bertrand [15,21], the first flush coefficient b is 0.185, and the b values corresponding to the 25/30, 30/30, 30/25, and 80/30 standards are the 0.862, 1.000, 1.159, 5.395, and corresponding judgments. Through the SPSS 24.0 software, the cumulative pollutant mass rate and cumulative sewage volume rate of each storm event were fitted according to Formula (2). Based on the location of different monitoring points and different storm events, a total of 63 storm events were used to calculate the pollutant first flush effect of the combined sewer system. According to the calculated value of b, the proportion of the corresponding range of each pollutant was obtained (Table 1).

The correlation coefficients R^2^ are all greater than 0.9, indicating that the fitted curve and the M(v) curve are in good agreement. Table 2 shows that the pollution first flush effect of the combined sewer system during wet weather in the study area is not universal, presenting different degrees of the first flush effect. TSS is the most common pollutant found in combined sewer sewage and has been recognized as the main indicator of stormwater quality [17]. Using TSS as a pollutant index, 12.69% of the storm events experienced a strong pollutant first flush effect, which may be due to heavy rainfall flushing the sewer and releasing the sewer sediments. However, 33.33–60.31% of the pollutant first flush effects of storm events are not obvious or remain non-existent. NH_4_^+^-N, TN, and TP show an observable first flush effect, whereas the first flush effect of COD is not obvious. Therefore, it is impossible to effectively control the combined sewer overflow by only treating sewage from the combined sewage system during the initial rainfall. The traditional rainwater storage mode for collecting rainfall at a depth of 3–5 mm cannot adapt to all situations; it is, therefore, necessary to develop a method to identify and collect polluted sewage.

In order to analyze the influence of rainfall variables on the water quality of the confluence system in the rainy season, this paper carried out a Pearson correlation analysis on five rainfall variables and pollutant concentrations, including rainfall, rainfall duration, average rainfall intensity, maximum 5 min intensity, and the antecedent dry period (Table 2). The results show that the concentration of TSS and TP in the combined sewer sewage in wet weather has a weak linear relationship with the rainfall depth and maximum rainfall intensity, which is similar to the study by Perera [17]. However, unlike the change in surface runoff pollutants, the correlation between the pollutants of the combined sewer system in wet weather and the two rainfall variables of rainfall depth and maximum rainfall intensity is weaker. This may be due to the complex composition of the combined sewer, which features the joint effect of surface pollutant scouring, sewer sediment scouring, and rainwater dilution.

### 3.3. Correlation Analysis of Pollutants and Turbidity

**Figure 3:**
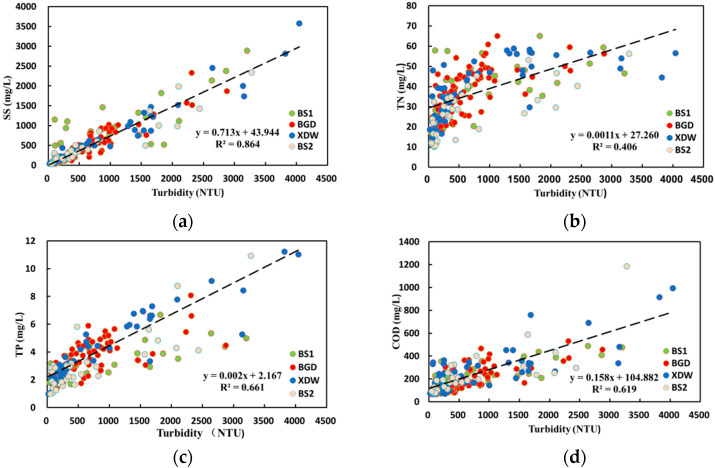
The linear fit relations of primary pollutants and turbidity at the four sampling points of combined sewer sewage in the southeast area of Beijing. (**a**): The linear fit relations of SS and turbidity; (**b**): The linear fit relations of TN and turbidity; (**c**): The linear fit relations of TP and turbidity; (**d**): The linear fit relations of COD and turbidity.

The correlation between TSS, TN, TP, COD, and real-time monitoring turbidity was analyzed. Figure 3a shows the correlation between TSS and turbidity at the four sampling points of combined sewer sewage in the southeast area of Beijing. TSS has a positive correlation with turbidity (R^2^ = 0.864, *p* < 0.05). Rossi [27] believes that during the heavy rain season, a large number of pollutants in CSOs are attached to suspended particles in water and that the concentration of total suspended solids is closely related to other pollutants. The TSS and turbidity have a high positive correlation, which indicates that the TSS concentration and other pollutants of the sewage from the combined sewer in wet weather can be expressed by an easily detectable turbidity value. Similarly, there is a positive correlation between TP and turbidity (R^2^ = 0.661, *p* < 0.01), but the correlation between TP and turbidity is lower than the correlation between TSS and turbidity. This may be due to the presence of soluble phosphate in the combined sewer sewage during wet weather. Moreover, the turbidity cannot fully represent the TP concentration. The positive correlation between TN and turbidity was weak (R^2^ = 0.406, *p* < 0.01). As described in Section 3.1, ammonium nitrogen accounts for 76.5–93.2% of the total nitrogen, and the concentration of NH_4_^+^-N fluctuates during dry weather. This may be due to the fact that in the study area, the main sources of ammonia nitrogen in the sewage from the combined sewer in wet weather are domestic sewage and industrial wastewater, not sewer sediments and surface pollutants. Therefore, the TN pollution contribution from surface runoff and sewer sediments caused by rainfall is small, and the correlation between turbidity and TN is poor. In addition, there is a positive correlation between COD and turbidity (R^2^ = 0.619, *p* < 0.01). Part of the COD is attached to the sewer sediments and suspended solids of the rainwater runoff, so it has a good linear relationship.

Concentrations of SS, TP, and COD in the combined sewer sewage during wet weather increased with an increase in sewage turbidity. Higher concentrations of SS, TP, and COD were observed in a range of turbidities. Real-time turbidity control is often used as a method to monitor the concentration of pollutants, which is closely related to the content of particulate matter in the wastewater. In this study, there was a good linear relationship between SS and turbidity in the sewage during wet weather. Part of TP and COD was also attached to these suspended particles, and TP, COD, and SS also showed a strong linear relationship, so the turbidity value was able to indicate the concentrations of SS, TP, and COD. SS is an important control parameter for river inflow pollution during wet weather. TP and COD are the main pollutants that cause eutrophication and the black odor of water bodies [28], so their concentrations can provide a general indication of the overall level of pollutants. Therefore, turbidity can be used as an identification parameter to selectively collect sewage from combined sewers in wet weather, allowing the stormwater detention tanks with a small capacity to be reasonably used.

### 3.4. Selective Collection Method of Sewage Based on Turbidity

Lacour [29] proposed that when there is a detention tank in the sewage network system, it has a higher potential for pollutant removal, which is also in line with the procedures developed by the German Association for Water, Wastewater, and Waste to determine the real-time turbidity control RTC potential of the system [30]. In order to effectively use the volume of the detention tank, turbidity is used to quickly identify and collect polluted sewage. Two steps are required for this process:

(1) Selecting control water quality parameters and determining the turbidity threshold;
(2) Regulate the fate of sewage according to the turbidity and water level of the effluent.

**Figure 4:**
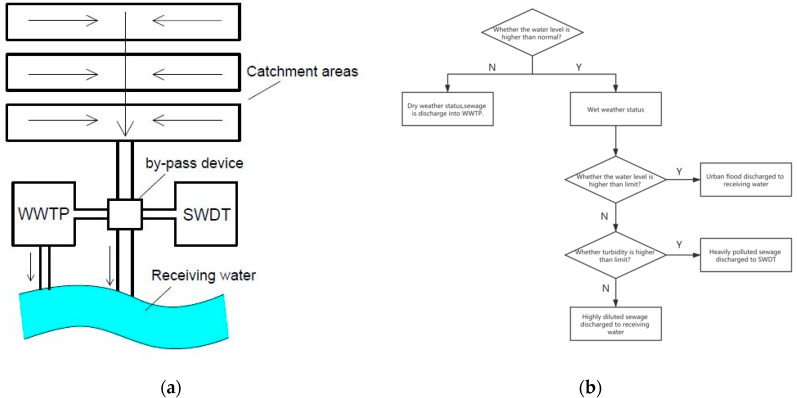
(**a**) Arrangement of the measurement equipment attached to the stormwater detention tank and (**b**) the process flow chart.

The ultimate goal of CSO control is to make the function of the receiving water body reach the standard. Therefore, no matter what water quality parameter is selected as the control standard, it is necessary to use the actual water quality as the goal, the overflow water quantity control of CSO in the basin as the means, and then finally determine the standard value and corresponding control scheme by comprehensively considering the cost and effect. Assuming that a certain river basin uses TSS as the control water quality parameter, the standard limit of TSS is set to 100 mg/L, and the regression equation in Section 3.3 is substituted, corresponding to a turbidity value of 78 NTU. When the turbidity of sewage in the combined sewer in wet weather is greater than 78 NTU, the sewage is considered to be seriously polluted and needs to be stored and treated. A liquid level meter and a turbidity sensor must be used together to judge the three states of dry weather, wet weather, and flood discharge to prevent domestic sewage with high turbidity from being discharged into the detention tank during dry weather. In addition, when the water level is higher than the limit, in order to prevent urban flooding, the inflowing water will be discharged into the river, even if the turbidity value exceeds the threshold. The identification equipment can be installed in the by-pass device, and the process flow chart is shown in Figure 4.

The biggest advantages of real-time turbidity control (RTC) are their short response time and adaptability to the randomness and spontaneity of rainfall events. In addition, the formulation of the CSO control plan in the planning and design phase should be based on the current baseline monitoring data and model evaluation (as a means). This model was continuously corrected and improved using the periodic monitoring data during the implementation of the CSO control project. Until the completion of all projects, the final effect of the CSO control plan can be determined through the monitoring data of water quality. Long-term monitoring with turbidity as the identification parameter is always performed to obtain the basic data.

## 4. Conclusions

Based on the adopted first flush detection approach, the pollutant first flush effect was not obvious or even non-existent for 47.62–69.84% of rainfall events, and the conventional rainwater storage model for stormwater detention tanks (collecting rainfall with a 3–5 mm depth) could not be adapted to all situations. This study proposes a method for selective wastewater collection based on real-time turbidity control. TSS, TP, and COD have a significant positive correlation with turbidity. The concentrations of TSS, TP, and COD can be determined by installing a turbidity meter in the facility. According to the online analysis, stormwater detention tanks can be fully used to collect heavily polluted sewage, which is conducive to reducing the negative impact of combined sewer overflow to the receiving water. In addition, turbidity can be used to monitor the water quality data of combined sewer systems over a long period of time.

## Figures and Tables

**Table 1:** The statistical characteristics of parameter b and the percentage of the b value of the pollutants (n = 63).

Pollutant	R^2^	Strong Distinctive First Flush (0 < b < 0.185)	Moderate First Flush (0.185 < b < 0.862)	Weak Distinctive First Flush (0.862 < b < 1)	First Flush Fails to Occur (1 < b)
Total suspended solids (TSS)	0.99	12.69%	26.98%	33.33%	26.98%
Chemical oxygen demand (COD)	0.99	7.94%	33.33%	39.68%	19.05%
NH_4_^+^-N	0.94	14.28%	15.87%	44.44%	25.40%
Total nitrogen (TN)	0.98	20.63%	31.74%	46.03%	1.59%
Total phosphorus (TP)	0.96	12.70%	38.09%	39.68%	9.52%

**Table 2:** Correlation analysis for rainfall variables and water quality parameters.

	TSS	NH_4_^+^-N	TN	TP	COD
Rainfall depth	0.245 *	/	/	0.188 *	/
Max rainfall intensity	0.291 *	0.107 *	/	0.296 *	/
Average rainfall intensity	/	0.263 *	0.214 *	0.201 *	/
Rain duration	/	/	/	/	/
Antecedent dry period	/	/	/	/	/

* represents statistical significance at a *p* < 0.05 level; / represents no correlation.
